# Antipsychotic Treatment-Associated Modulation of ABC Transporter Genes (ABCC1, ABCB1, and ABCA2) in Schizophrenia: A Longitudinal Expression Study

**DOI:** 10.3390/genes16121471

**Published:** 2025-12-09

**Authors:** Filiz Ekim Çevik, Esra Guzel Tanoglu, Kadriye Nur Cakmur, Muhammed Fevzi Esen, Fatma Rumeysa Uzun, Murat Erkiran

**Affiliations:** 1 Department of Medical Sciences, Institute of Forensic Sciences and Legal Medicine, Istanbul University-Cerrahpasa, Istanbul 34500, Turkey; 2 Department of Molecular Biology and Genetics, Institution of Hamidiye Medical Sciences, University of Health Sciences Turkey, Istanbul 34668, Turkey; 3 Experimental Medicine Research and Application Center, University of Health Sciences Turkey, Istanbul 34662, Turkey; 4 Department of Psychiatry, Bakirkoy Training and Research Hospital for Psychiatry, Neurology and Neurosurgery, Istanbul 34147, Turkey; 5 Health Information System, University of Health Sciences Turkey, Istanbul 34093, Turkey

**Keywords:** schizophrenia, ABCC1, ABCB1, ABCA2, gene expression, ABC transporters, pharmacogenomics

## Abstract

Background: ATP-binding cassette (ABC) transporters regulate xenobiotic efflux, oxidative stress responses, and blood–brain barrier (BBB) homeostasis. Dysregulation of transporters such as ABCC1, ABCB1, and ABCA2 has been linked to neuropsychiatric disorders, yet their expression patterns in schizophrenia and their modulation by antipsychotic treatment remain unclear. This study investigated longitudinal changes in the expression of these genes in schizophrenia patients before and after antipsychotic therapy, compared with healthy controls. Methods: Sixty individuals with schizophrenia and sixty matched healthy controls were included. Serum samples were obtained from patients during the acute pre-treatment phase and after clinical improvement following antipsychotic therapy. Gene expression of ABCC1, ABCB1, and ABCA2 was measured by RT-qPCR (normalized to ACTB). Log2 fold-change (log2FC) values relative to controls were calculated. Group differences were assessed with Mann–Whitney U and Wilcoxon signed-rank tests, and associations with clinical severity were analyzed using correlations with Positive and Negative Syndrome Scale (PANSS) scores. Results: In the acute phase, ABCC1 and ABCB1 expression were significantly downregulated in schizophrenia compared with controls (both *p* < 0.001). Antipsychotic treatment produced significant increases in both genes, though expression remained below control levels. ABCA2 showed no baseline differences but exhibited marked upregulation after treatment (*p* < 0.001). Higher baseline ABCC1 expression was associated with greater pre-treatment symptom severity, whereas higher baseline ABCB1 expression was associated with, rather than predicted, poorer clinical improvement. No significant correlations were found for ABCA2. Conclusions: These findings demonstrate distinct, gene-specific alterations in ABC transporter expression in schizophrenia. ABCC1 and ABCB1 appear suppressed during acute illness and partially restored with antipsychotic therapy, while ABCA2 shows a strong treatment-related upregulation. ABC transporter expression—particularly ABCB1—may provide preliminary molecular insight into treatment-related variability, although biomarker utility cannot be established from the present data. Longitudinal pharmacogenomic studies are needed to clarify their clinical relevance.

## 1. Introduction

Schizophrenia is a severe psychiatric disorder characterized by chronic neurobiological dysregulation and heterogeneous patterns of treatment response [1]. Although antipsychotic medications are the cornerstone of management, a considerable proportion of patients exhibit inadequate or only partial response to standard therapies, contributing to the ongoing challenge of treatment resistance [1,2,3]. Increasing evidence suggests that pharmacokinetic mechanisms—particularly drug transport across the blood–brain barrier (BBB)—play a key role in shaping antipsychotic efficacy and individual variability in treatment outcomes [4,5,6].

ATP-binding cassette (ABC) transporters represent a major superfamily of efflux pumps involved in xenobiotic clearance, oxidative stress regulation, and intracellular drug disposition. Among these, ABCB1 (P-glycoprotein) and ABCC1 (MRP1) are functionally active at the BBB, where they modulate the penetration and distribution of several psychotropic medications within the central nervous system (CNS) [7,8,9]. Beyond their pharmacokinetic influence, both ABCB1 and ABCC1 participate in glutathione-dependent detoxification and oxidative stress responses—pathways strongly implicated in the neurobiology of schizophrenia. ABCA2, although less extensively characterized, is highly expressed in oligodendrocyte-rich and glial regions of the brain. This transporter contributes to intracellular lipid trafficking, myelin integrity, and membrane homeostasis, and alterations in its function have been linked to neurodevelopmental abnormalities and dysregulated cellular metabolism [10,11]. These mechanisms suggest a plausible role for ABCA2 in schizophrenia-related neural processes.

A growing body of research has examined the interaction between antipsychotic medications and ABC transporters. Experimental and clinical studies indicate that antipsychotics may influence transporter activity or gene expression, thereby affecting the accumulation of drugs within the CNS [7,12,13]. Pharmacogenomic findings further show that functional variants in ABC transporter genes contribute to interindividual variability in treatment response, therapeutic plasma concentrations, and susceptibility to adverse effects [9,12].

Despite these advances, human studies evaluating antipsychotic treatment-associated transcriptional changes in ABC transporters—particularly ABCC1, ABCB1, and ABCA2—remain scarce. Existing studies are mostly cross-sectional, focus on genetic polymorphisms rather than expression levels, or rely primarily on in vitro or animal models. Thus, the dynamic transcriptional responses of these transporters during the course of treatment are not well understood.

The present study aims to address this gap by examining the longitudinal expression patterns of ABCC1, ABCB1, and ABCA2 in individuals with schizophrenia before and after antipsychotic treatment, and by comparing these patterns with those of healthy controls. By integrating molecular expression data with clinical symptom severity, the study seeks to elucidate the potential role of ABC transporters in the biological mechanisms underlying antipsychotic treatment response.

## 2. Methods

### 2.1. Participants and Sample Collection

A total of 120 participants were recruited between 2022 and 2023 from the psychiatry clinics of Prof. Dr. Mazhar Osman Bakırköy Mental Health and Neurological Diseases Training and Research Hospital. The sample comprised 60 individuals diagnosed with schizophrenia and 60 healthy control participants. Serum samples were obtained from schizophrenia patients during the acute psychotic episode (pre-treatment; *n* = 60) and again following clinical improvement after antipsychotic therapy (post-treatment; *n* = 60). Gene expression analyses of ABCC1, ABCB1, and ABCA2 were performed in all serum samples from patients and controls.

Demographic and clinical characteristics were recorded for all participants. Schizophrenia patients were assessed using the Positive and Negative Syndrome Scale (PANSS) during both the psychotic exacerbation phase and the early remission period. Eligibility criteria included: age 18–65 years, ability to provide informed consent, literacy, and absence of intellectual disability. Individuals in the schizophrenia group met DSM-5 diagnostic criteria, had no systemic or neurological medical illness, had no history of alcohol or substance use disorder, and had not received antipsychotic treatment for at least two months prior to clinical admission.

Healthy controls met the same general inclusion criteria and additionally were confirmed to have no lifetime or current psychiatric disorder based on a structured clinical interview. All participants provided written and verbal informed consent after receiving detailed information about the study procedures. The study protocol was approved by the Ethics Committee of the University of Health Sciences (No: 02.09.2022/20.09) and conducted in accordance with the Declaration of Helsinki.

### 2.2. RNA Extraction

Total RNA was isolated from serum using the One Step-RNA Reagent (Bio Basic, Markham, ON, Canada) following the manufacturer’s instructions. The concentration and purity of extracted nucleic acids were assessed using a BioSpec-nano spectrophotometer (Shimadzu Biotech, Kyoto, Japan).

### 2.3. cDNA Synthesis and Real-Time PCR

cDNA synthesis was executed with Step One Plus (Applied Biosystem, Foster City, CA, USA) using 10 ng cDNA. The RT-qPCRs were performed in duplicate for each of the three groups. Quantitative real-time polymerase chain reaction (qRT-PCR) was carried out using Blastaq™ 2X qPCR Master Mix (Applied Biological Materials, Richmond, BC, Canada) on a Bio-Rad CFX96 Real-Time System thermal cycler (Bio-Rad, Hercules, CA, USA). The qRT-PCR cycling conditions were as follows: an initial incubation at 50 °C for 2 min and 95 °C for 10 min, followed by 40 cycles of denaturation at 95 °C for 15 s and annealing/extension at 60 °C for 1 min. The gene expression was normalized to ACTB as a reference gene using related primers (ABCC1: F-TCAGCCAGAAAATCCTCCAC- & R-GGCACCATGAGGACCATC, ABCB1-1: F-CCCATCATTGCAATAGCAGG & R-GTTCAAACTTCTGCTCCTGA, ABCA2: F-AGATG GACAAGATGATCGAG & R-GCTTGTACTTCAGGATGAGG). The mean of ΔCT for each group was calculated, and eventually, the relative expression of each gene was estimated by ratio, i.e., 2^−ΔΔCt^ (fold change) as described by Livak [PMID: 11846609].

### 2.4. Statistical Analysis

A power analysis was performed using G*Power 3.1.9.2 to determine the minimum sample size required to detect significant differences in log2 fold change (log2 FC) expression. Based on Cohen’s d = 0.4 with α = 0.05 and a power of 0.80, the minimum total sample size was calculated as 52. To ensure robust detection of effects and account for potential covariates, 60 participants per group were included in the sample (healthy controls, pre-treatment patients, and post-treatment patients).

Statistical analyses were performed using SPSS version 26.0 (IBM Corp., Armonk, NY, USA). The Shapiro–Wilk test confirmed significant deviations from normality for all genes across groups (ABCC1: *p* = 0.003; ABCB1: *p* < 0.001; ABCA2: *p* < 0.001). Consequently, non-parametric statistical tests were employed to compare the groups due to data distribution. Mann–Whitney U test was employed to assess differences between the control and patient groups. The Wilcoxon signed-rank test was utilized for comparisons between pre-treatment and post-treatment conditions within the same patient group.

## 3. Results

The sociodemographic characteristics of participants with schizophrenia and healthy controls are presented in Table 1. The mean age of the schizophrenia participants was 40.18 years, while the healthy control participants had a mean age of 37.12 years, with similar age distributions across males and females in both groups. Marital status and education levels also showed comparable patterns. Most individuals in the schizophrenia group were single or had lower levels of formal education, whereas the healthy control group included a higher proportion of married and undergraduate-educated individuals. Overall, the demographic profiles indicated adequate separation of clinical and control groups, with balanced gender distributions.

Table 2 summarizes the PANSS scores of participants with schizophrenia by gender. Pre-treatment PANSS total scores were numerically similar between males (133.79) and females (126.19), and a comparable pattern was observed in the post-treatment period (males: 69.89; females: 63). These descriptive findings indicate that both baseline symptom severity and treatment-related improvement were consistent across genders.

Gene expression analyses were conducted using log2 fold-change (log2FC) values relative to the healthy control group. The statistical significance threshold was set at *p* = 0.05, and all *p*-values were adjusted for multiple comparisons using the Benjamini–Hochberg procedure.

**Figure 1:**
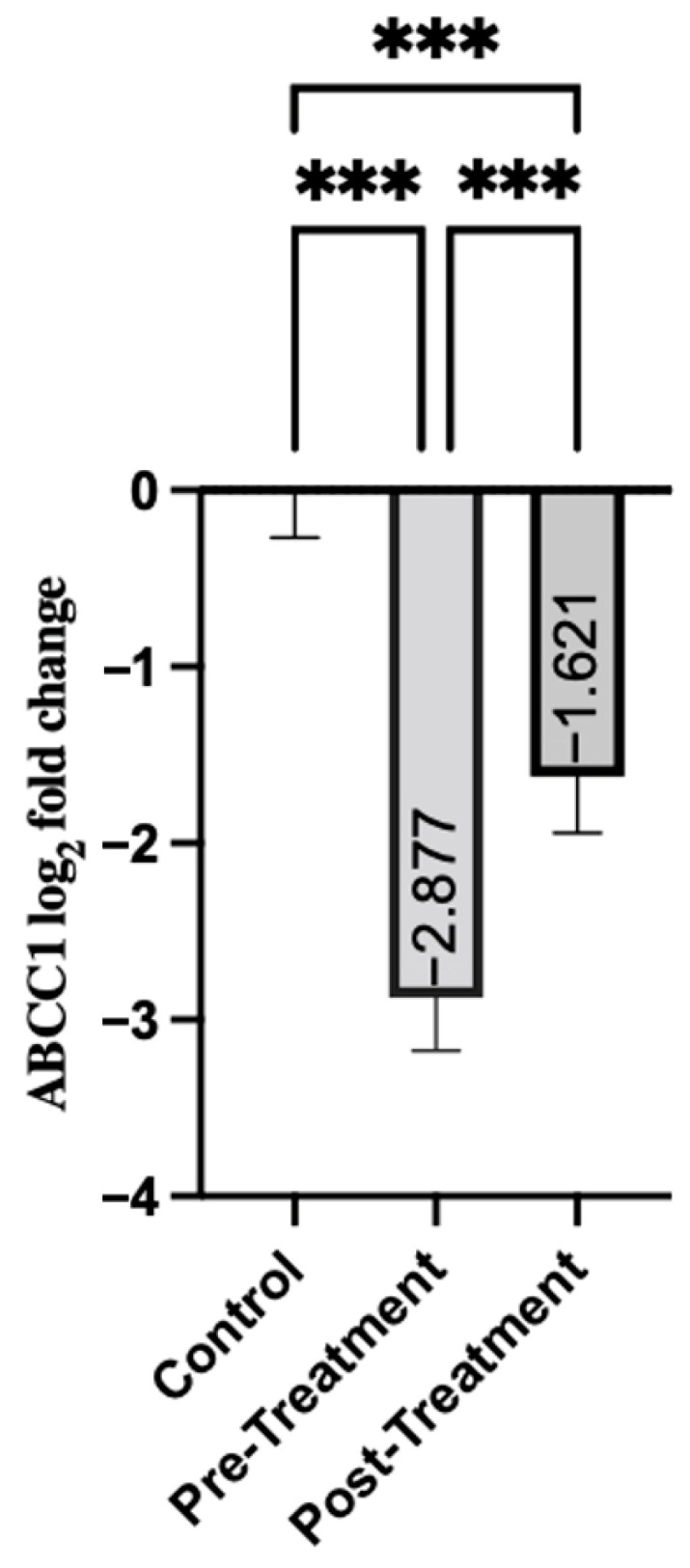
Comparison of Groups for ABCC1. Control samples are centered at log2FC = 0 by definition. Mann–Whitney U test was used for comparisons between control and pre-treatment/post-treatment. The Wilcoxon test was used for paired comparisons. *** *p* < 0.001.

Analysis of the ABCC1 gene expression revealed differences in people with schizophrenia relative to healthy controls. The patients before treatment had significant downregulation, with a mean expression of −2.88 (SD = 2.31), as shown in Table 3. ABCC1 expression increased to −1.62 in the post-treatment group (SD = 2.49). Although this post-treatment level was much lower than that of the control group (*p* < 0.001), the increase compared to pre-treatment levels was statistically significant (*p* < 0.001). This may indicate that the treatment led to a measurable increase in ABCC1 expression (Figure 1).

**Table 3:** Descriptive statistics of ABCC1.

Statistics	Control	Pre-Treatment	Post-Treatment
n	60	60	60
Minimum	−4.15	−8.96	−6.19
25% Percentile	−1.02	−4.28	−3.12
Median	0.00	−3.26	−2.02
75% Percentile	0.672	−1.12	−0.499
Maximum	6.38	2.33	5.86
Mean	0.00	−2.88	−1.62
Std. Deviation	2.07	2.31	2.49

**Figure 2:**
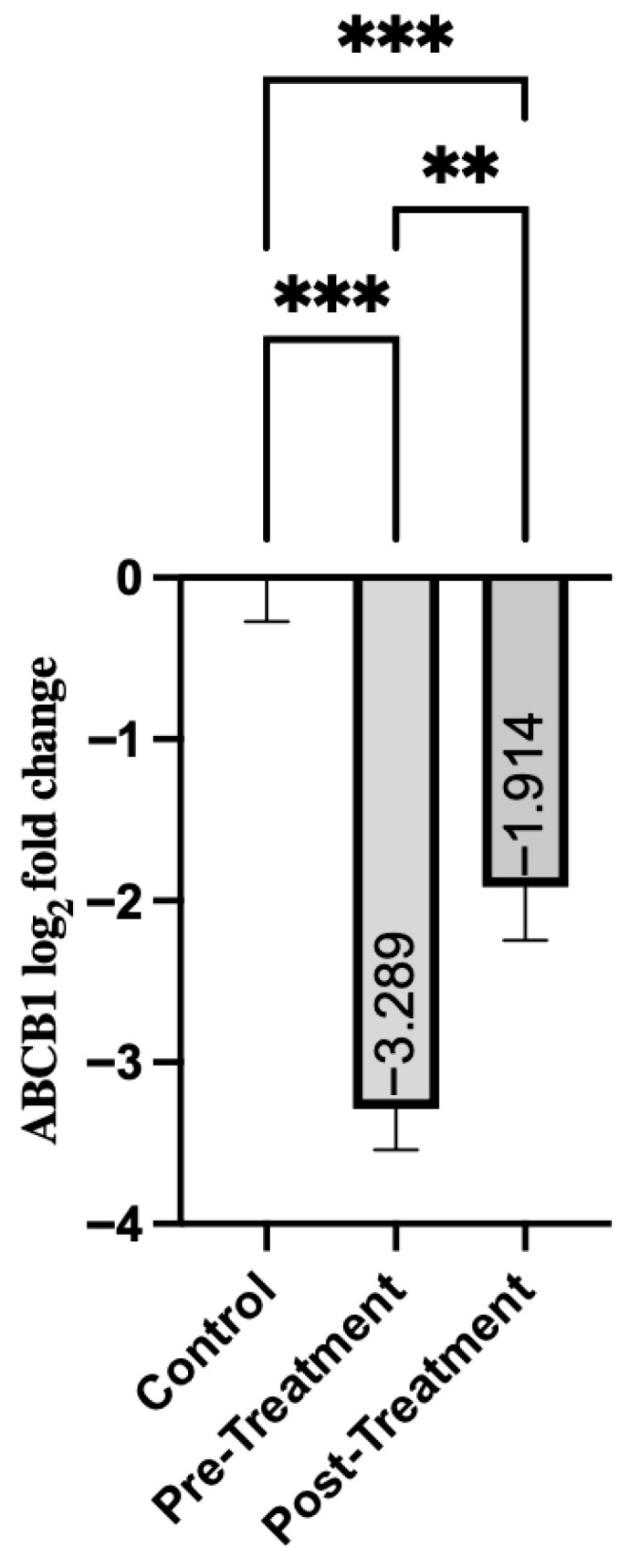
Comparison of Groups for ABCB1. Control samples are centered at log2FC = 0 by definition. Mann–Whitney U test was used for comparisons between control and pre-treatment/post-treatment groups. The Wilcoxon test was used for paired comparisons. ** *p* < 0.01, *** *p* < 0.001.

ABCB1 gene expression showed a similar pattern, with significant differences between patients with schizophrenia and the control group. Pre-treatment group exhibited a significant decrease in ABCB1 expression (mean = −3.29, SD = 1.95) (Table 4). This suggests a downregulation of the disease state. As shown in Figure 2, post-treatment expression showed a modest increase (mean = −1.91, SD = 2.57). That is, while it remained significantly lower than in the control group (*p* < 0.001), it showed an improvement over pre-treatment levels (*p* = 0.008).

**Table 4:** Descriptive statistics of ABCB1.

Statistics	Control	Pre-Treatment	Post-Treatment
n	60	60	60
Minimum	−6.01	−7.20	−6.59
25% Percentile	−0.778	−4.59	−3.53
Median	−0.125	−3.56	−2.49
75% Percentile	0.415	−2.27	−0.431
Maximum	5.74	1.68	5.44
Mean	0.00	−3.29	−1.91
Std. Deviation	2.091	1.951	2.571

**Figure 3:**
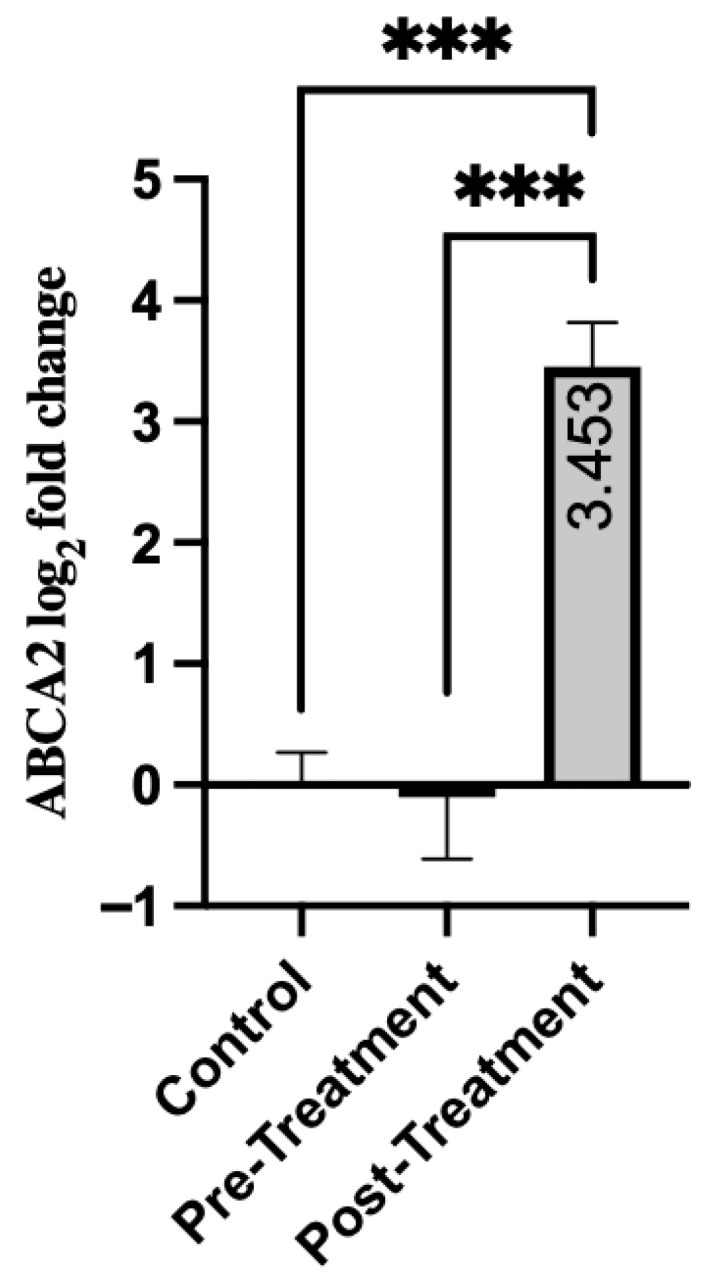
Comparison of Groups for ABCA2. Control samples are centered at log2FC = 0 by definition. Mann–Whitney U test was used for comparisons between the control and pre-/post-treatment groups. The Wilcoxon signed-rank test was used for paired comparisons. *** *p* < 0.001.

Expression of the ABCA2 gene showed a significant response to treatment in patients. As shown in Table 5, expression levels were relatively low in the pre-treatment period (mean = −0.102, SD = 3.94). After treatment, there was a significant increase with a mean of 3.45 (SD = 2.81). There was a statistically significant difference in pre-treatment and post-treatment scores (*p* < 0.001). Moreover, both pre-treatment and post-treatment groups showed significant differences from the healthy control group. There was no significant difference between the pre-treatment and control groups (*p* = 0.428) (Figure 3). However, when the post-treatment group was compared with the control group, a highly significant increase was noted (*p* < 0.001). The data suggest that ABCA2 is markedly upregulated by treatment in schizophrenia, which may indicate gene-specific pharmacological activation or a compensatory regulatory mechanism.

Analysis of ABC transporters in patients revealed different expression patterns. Prior to treatment, ABCC1 and ABCB1 exhibited significant downregulation compared to healthy controls (9.5-fold and 11.8-fold reduction, respectively). Treatment significantly reduced these impairments (4.0-fold and 2.1-fold improvement; *p* = 0.008). In contrast, ABCA2 showed no dysregulation at baseline (*p* = 0.428). However, a treatment-induced upregulation was observed (5.7-fold increase compared to controls, *p* < 0.001).

According to Table 6, the expression level of ABCC1 in the pre-treatment group showed a significant positive correlation with pre-treatment PANSS Score (r = 0.326, *p* = 0.011). The correlation between pre-treatment ABCC1 level and post-treatment PANSS Score was positive but not statistically significant (r = 0.162, *p* = 0.216). Similarly, the correlation involving the post-treatment ABCC1 expression was also not significant (r = −0.148, *p* = 0.259).

ABCB1 (in the pre-treatment group) showed a significant positive correlation with post-treatment PANSS Score (r = 0.351, *p* = 0.006). This may be a notable finding indicating that higher baseline expression of ABCB1 is associated with poor treatment outcomes (higher symptom severity) after the treatment period. For ABCA2, none of the measured correlations reached statistical significance (all *p* > 0.05). This suggests that there is no clear evidence of a relationship between ABCA2 gene expression and symptom severity before or after treatment.

## 4. Discussion

The present study provides comprehensive insights into the molecular and clinical characteristics of schizophrenia by integrating sociodemographic patterns, symptom severity indices, and expression profiles of key ATP-binding cassette (ABC) transporters. The sociodemographic features of our schizophrenia cohort—particularly lower marital rates and reduced levels of educational attainment—mirror well-established epidemiological trends. These patterns have consistently been linked to the chronicity of illness, functional impairment, and pervasive social disadvantage experienced by individuals with schizophrenia [14,15,16,17,18]. The largely comparable age and gender distributions between patient and control groups reinforce the internal validity of the study by reducing demographic confounding in subsequent analyses.

From a clinical standpoint, symptom severity as assessed through the PANSS instrument demonstrated relatively parallel pre- and post-treatment trajectories between males and females. This suggests that gender did not exert a substantial influence on baseline symptomatology or short-term treatment responsiveness within this sample. Although sex-specific differences—including earlier onset among males, divergent neurodevelopmental risk pathways, and differential long-term outcomes—have been widely documented [19,20], several controlled studies have shown that such divergences may attenuate during comparable phases of illness or within short-term therapeutic windows [21]. The similarity of symptom improvement across genders in our study, therefore, strengthens the interpretation that molecular findings—particularly differences in transporter gene expression—are unlikely to be driven by systematic gender effects.

A major molecular contribution of the study derives from the analysis of ABCC1 gene expression. Our findings revealed significant downregulation of ABCC1 in the acute, antipsychotic-free phase of schizophrenia. However, because ABCC1 levels were quantified in peripheral serum rather than central nervous system tissue, any mechanistic interpretation regarding BBB transporter function should be made cautiously. This pattern may be broadly compatible with and aligns with emerging mechanistic frameworks that posit BBB dysfunction as a central pathophysiological feature of schizophrenia, although our data do not directly address BBB integrity or CNS transporter regulation. Theoretical and translational models further suggest that neurovascular unit dysfunction and BBB hyperpermeability play critical roles in disrupting endothelial transporter regulation during acute psychosis [22]. Chronic neuroinflammation, oxidative stress, pericyte loss, and endothelial signaling abnormalities have all been shown to impair BBB integrity and alter the expression of efflux transporters, including ABCC family members [2,23]. While these mechanisms provide useful context, our findings represent peripheral molecular signatures rather than direct evidence of altered BBB transporter activity. Suppressed ABCC1 activity may result in diminished clearance of inflammatory mediators, neurotoxic metabolites, and xenobiotics from the brain; however, the present serum-based analysis does not include protein-level or pharmacokinetic assessments required to evaluate such effects directly. Consistent with this mechanistic view, transcriptomic studies have demonstrated that individuals with schizophrenia exhibit widespread dysregulation in immune and inflammatory pathways, including those associated with molecular efflux, endothelial function, and BBB maintenance [24,25]. Our results may therefore reflect peripheral changes that align with these pathways, rather than direct measures of BBB dysfunction.

The significant increase in ABCC1 expression following antipsychotic treatment observed in our cohort suggests a peripheral molecular shift associated with clinical stabilization, rather than a partial restoration of efflux function. Although post-treatment levels did not normalize to those of healthy controls, the measurable upward shift reflects a treatment-related adjustment in expression rather than biologically meaningful recovery of BBB-related homeostasis. Findings from the Accelerating Medicines Partnership (AMP) Schizophrenia Program further corroborate the dynamic nature of inflammatory and transporter-related gene networks, showing that psychosis risk states are associated with robust inflammatory transcriptional signatures that may shift toward partial attenuation following effective treatment [26]. Additionally, pharmacogenetic influences may modulate ABCC1 expression; variants in ABC transporter genes have been shown to impact antipsychotic transport efficiency and CNS bioavailability, although serum-based gene expression cannot directly inform CNS availability [12]. Preclinical studies further reveal that antipsychotics differentially bind to transporter proteins, including P-glycoprotein and related efflux pumps, which may contribute to compensatory regulatory changes, although such mechanisms were not assessed in the present study [13]. Together, these contextual factors provide a broader interpretive framework for understanding the post-treatment increase in ABCC1 expression as a peripheral molecular adaptation rather than direct evidence of restored central efflux capacity.

A similarly nuanced pattern emerged for ABCB1. In the untreated state, ABCB1 expression was markedly reduced, although this peripheral finding should not be taken as direct evidence of multidimensional impairments in BBB efflux systems. ABCB1/P-glycoprotein is responsible for controlling the entry of numerous psychotropic drugs, endogenous metabolites, and inflammatory molecules into the CNS. While downregulation of this transporter has been hypothesized to influence the accumulation of inflammatory mediators and altered neurotransmission, such effects cannot be inferred from serum mRNA levels alone. Mechanistic reviews highlight that cytokine-driven suppression, oxidative damage, and endothelial distress—common features of schizophrenia—can significantly inhibit ABCB1 transcription [2,23,24]. Transcriptomic and postmortem studies reinforce this association by demonstrating downregulation of barrier-related genes in schizophrenia, particularly in frontal cortical regions implicated in psychosis [25,27]. Our findings may therefore reflect peripheral expression patterns that align with these broader molecular signatures, rather than direct indicators of altered BBB transporter function.

Interestingly, and clinically relevant, the partial restoration of ABCB1 expression following antipsychotic treatment did not fully normalize transporter levels. This post-treatment change is consistent with longitudinal biomarker research demonstrating that neuroinflammatory load diminishes during treatment, potentially contributing to peripheral transcriptional adjustments rather than directly indicating improved BBB endothelial signaling [26]. The more notable finding is the significant positive correlation between pre-treatment ABCB1 expression and post-treatment PANSS scores; however, this association should be interpreted cautiously, as its modest strength precludes predictive or causal conclusions regarding treatment response. This interpretation aligns with established pharmacokinetic evidence demonstrating that elevated ABCB1/P-glycoprotein activity at the blood–brain barrier enhances the efflux of antipsychotic medications, thereby limiting their central penetration and reducing therapeutic efficacy [28], although peripheral mRNA levels cannot directly inform CNS drug transport or BBB efflux capacity. Beyond its efflux function, ABCB1 activity is tightly linked to membrane lipid composition and microdomain organization, which can influence transporter conformation and substrate affinity. ABC transporters, including ABCB1, operate within lipid-rich regions of the membrane, where cholesterol dynamics and lipid–protein interactions modulate transporter behavior and drug transport efficiency [29]. While these mechanisms provide meaningful biological context, they were not directly evaluated in the present study, and the observed associations should therefore be viewed as peripheral molecular patterns rather than evidence of increased drug-efflux capacity or membrane-related resistance mechanisms in vivo.

By contrast, ABCA2 demonstrated a distinctly different expression profile. Unlike ABCC1 and ABCB1, ABCA2 showed no significant dysregulation at baseline, suggesting that this transporter is not directly suppressed during acute illness. However, its expression increased significantly following antipsychotic treatment, exceeding control levels. This pattern may reflect a treatment-associated peripheral metabolic adjustment rather than a pathophysiological deficit. Recent lipidomic studies have demonstrated marked perturbations in cholesterol metabolism, phospholipid composition, and membrane homeostasis in schizophrenia [30]. Given its role in intracellular cholesterol trafficking, lysosomal lipid transport, and myelin-associated processes, ABCA2 is considered responsive to metabolic and pharmacological stimuli rather than an indicator of acute inflammatory stress [28]. The pronounced post-treatment upregulation of ABCA2 observed in our cohort may therefore represent a peripheral adaptive response during antipsychotic exposure, although specific lipid-stabilizing or metabolic mechanisms cannot be inferred from mRNA expression alone.

The correlation analyses reinforce these mechanistic distinctions. Pre-treatment ABCC1 positively correlated with baseline PANSS scores, although this association should be interpreted cautiously, as peripheral expression may reflect broader inflammatory activity rather than specific BBB-related processes. Conversely, higher baseline ABCB1 expression was associated with poorer post-treatment outcomes, but this modest correlation does not support predictive or causal interpretation, and the relationship may be conceptually compatible with pharmacokinetic evidence suggesting that elevated ABCB1/P-glycoprotein activity at the blood–brain barrier can reduce central antipsychotic exposure, although peripheral mRNA levels cannot directly inform CNS penetration or efflux capacity.

ABCA2, however, demonstrated no correlation with clinical severity at either time point, consistent with its role as a treatment-responsive peripheral metabolic marker rather than a transporter directly linked to psychopathology. Collectively, these findings underscore the potential gene-specific heterogeneity of ABC transporter biology in schizophrenia: ABCC1 may relate to inflammatory variability, ABCB1 may reflect pharmacodynamic differences, and ABCA2 may index treatment-associated metabolic adaptations.

This study has several limitations that should be considered when interpreting the findings. First, the sample size, although adequate for medium effect detection, limits broader generalizability. Second, gene expression was assessed in peripheral blood rather than brain tissue; while peripheral markers can provide valuable insights into systemic inflammatory and transporter-related processes, they do not directly reflect region-specific transporter activity or blood–brain barrier regulation, and serum mRNA cannot be used to infer CNS efflux function. Future studies incorporating multimodal imaging, CSF-based biomarkers, or cell-specific approaches (e.g., induced pluripotent stem cell-derived endothelial models) would help clarify these mechanisms. Third, we did not evaluate functional polymorphisms (SNPs) in ABC transporter genes, which may influence expression patterns or treatment response; incorporating pharmacogenetic analyses in future studies would strengthen mechanistic interpretation.

Our findings demonstrate that ABC transporters exhibit distinct peripheral regulatory patterns in schizophrenia, with ABCC1 and ABCB1 potentially relating to inflammatory- and pharmacodynamic variability, while ABCA2 may reflect a treatment-associated metabolic adjustment rather than a pathophysiological deficit. These transporter-specific trajectories may offer preliminary molecular insight, although definitive biomarker utility cannot be established from the present data. Future studies integrating pharmacogenetic profiling, direct assessments of BBB integrity, and longitudinal multimodal approaches are warranted to clarify the functional implications of transporter regulation. Such advances may help inform more precise and personalized therapeutic strategies in schizophrenia.

## Figures and Tables

**Table 1:** Sociodemographic characteristics of participants with schizophrenia and healthy controls by gender.

	Patient			Control		
Variables	Male (n = 39) (Mean)	Female (n = 21) (Mean)	Total (n = 60) (Mean)	Male (n = 35)	Female (n = 25)	Total (n = 60)
Age (years)	39.49	41.48	40.18	36.5	38	37.12
Marital status						
married	6	9	15	28	20	48
single	32	8	40	5	4	9
divorced	1	4	5	3	1	4
Education level						
primary	10	7	17	-	-	-
secondary	15	3	18	-	-	-
collage	10	6	16	10	5	15
undergraduate	4	5	9	25	20	45

**Table 2:** Mean PANSS scores of participants with schizophrenia by gender.

Patient	Male (n = 39) (Mean)	Female (n = 21) (Mean)	Total (n = 60) (Mean)
Mean PANSS (Pre-treatment)	133.79	126.19	131.13
Mean PANSS (Post-treatment)	69.89	63	67.44

**Table 5:** Descriptive statistics of ABCA2.

Statistics	Control	Pre-Treatment	Post-Treatment
n	60	60	60
Minimum	−3.13	−7.06	−1.21
25% Percentile	−0.960	−3.23	1.68
Median	−0.394	−0.904	2.52
75% Percentile	0.677	3.46	4.57
Maximum	5.20	10.2	11.1
Mean	0.00	−0.102	3.45
Std. Deviation	2.05	3.94	2.81

**Table 6:** Correlation analyses between ABCC1, ABCB1, and ABCA2 expression levels and PANSS scores.

Genes	Groups	Pre-Treatment PANSS Score	Post-Treatment PANSS Score
ABCC1	Pre-Treatment	0.326 (0.011)	0.162 (0.216)
	Post-Treatment	0.130 (0.322)	−0.148 (0.259)
ABCB1	Pre-Treatment	0.166 (0.206)	0.351 (0.006)
	Post-Treatment	0.113 (0.390)	−0.089 (0.498)
ABCA2	Pre-Treatment	0.164 (0.211)	0.135 (0.303)
	Post-Treatment	0.148 (0.259)	−0.039 (0.766)

Note. Values represent the Pearson correlation coefficient (r) between gene expression and the PANSS Score. *p*-values indicating the statistical significance of the correlation are provided in parentheses.

## Data Availability

The data presented in this study are available from the corresponding author upon reasonable request. The data are not publicly available due to ethical and privacy restrictions.data presented in this study are available upon request from the corresponding author.
